# Acute Cellular and Functional Changes With a Combinatorial Treatment of Ion Channel Inhibitors Following Spinal Cord Injury

**DOI:** 10.3389/fnmol.2020.00085

**Published:** 2020-06-25

**Authors:** Ryan L. O’Hare Doig, Sreya Santhakumar, Brooke Fehily, Sushmitha Raja, Tanya Solomon, Carole A. Bartlett, Melinda Fitzgerald, Stuart I. Hodgetts

**Affiliations:** ^1^Experimental and Regenerative Neurosciences, School of Biological Sciences, The University of Western Australia, Crawley, WA, Australia; ^2^Experimental and Regenerative Neurosciences, School of Human Sciences, The University of Western Australia, Crawley, WA, Australia; ^3^Neil Sachse Centre for Spinal Cord Research, South Australian Health and Medical Research Institute, Adelaide, SA, Australia; ^4^Adelaide Spinal Research Group, Faculty of Health and Medical Sciences, The University of Adelaide, Adelaide, SA, Australia; ^5^Perron Institute for Neurological and Translational Science, Nedlands, WA, Australia; ^6^Curtin Health Innovation Research Institute, Curtin University, Nedlands, WA, Australia

**Keywords:** spinal cord injury, secondary degeneration, ion channel inhibitors, glia, calcium (Ca^2+^), oxidative stress

## Abstract

Reducing the extent of secondary degeneration following spinal cord injury (SCI) is necessary to preserve function, but treatment options have thus far been limited. A combination of the ion channel inhibitors Lomerizine (Lom), YM872 and oxATP, to inhibit voltage-gated Ca^2+^ channels, Ca^2+^ permeable AMPA receptors, and purinergic P2X_7_ receptors respectively, effectively limits secondary consequences of injury in *in vitro* and *in vivo* models of CNS injury. Here, we investigated the efficacy of these inhibitors in a clinically relevant model of SCI. Fischer (F344) rats were subjected to a moderate (150 kD) contusive SCI at thoracic level T10 and assessed at 2 weeks or 10 weeks post-injury. Lom was delivered orally twice daily and YM872 and oxATP were delivered *via* osmotic mini-pump implanted at the time of SCI until 2 weeks following injury. Open field locomotion analysis revealed that treatment with the three inhibitors in combination improved the rate of functional recovery of the hind limb (compared to controls) as early as 1-day post-injury, with beneficial effects persisting to 14 days post-injury, while all three inhibitors were present. At 2 weeks following combinatorial treatment, the functional improvement was associated with significantly decreased cyst size, increased immunoreactivity of β-III tubulin^+ve^ axons, myelin basic protein, and reduced lipid peroxidation by-products, and increased CC1^+ve^ oligodendrocytes and NG2^+ve^/PDGFα^+ve^ oligodendrocyte progenitor cell densities, compared to vehicle-treated SCI animals. The combination of Lom, oxATP, and YM872 shows preclinical promise for control of secondary degeneration following SCI, and further investigation of long-term sustained treatment is warranted.

## Introduction

Spinal cord injury (SCI) is a seriously debilitating event that can quickly lead to paralysis or even death, with a large physical, emotional, and socioeconomic burden (Ditunno and Formal, 1994). In the United States alone, it is estimated that the annual incidence of SCI injury is 17,000 each year, with approximately 282,000 persons currently living with a SCI (White and Black, 2016). Following SCI, there is an injury severity dependent disruption of axonal pathways (Fehlings and Tator, 1995) impairing motor, sensory and autonomic function at and below the site of injury. Although a significant understanding of the etiology and pathophysiology of SCI has been attained, an effective therapeutic treatment strategy for SCI remains elusive.

When the central nervous system (CNS) is contused, or damaged by a penetrative or compressive force, a plethora of molecular and cellular cascades described as secondary degeneration, exacerbate neurological damage, and functional impairment. The acute phase of secondary degeneration in the CNS can occur minutes to weeks following injury, with the chronic phase appearing from within months to a year (Dihné et al., 2001; Nashmi and Fehlings, 2001; Chen et al., 2003; Fitzgerald et al., 2010; Payne et al., 2012; Petersen et al., 2012). It is understood that the biochemical events associated with secondary degeneration include, but are not limited to glutamate excitotoxicity (Szydlowska and Tymianski, 2010; Tsutsui and Stys, 2013) disruptions of ionic balance of K^+^, Na^+^, and Ca^2+^ (Choi, 1987; Stys, 2004) free radical formations and lipid peroxidation (Kontos and Wei, 1986; Liu et al., 2004; Vaishnav et al., 2010) with apoptosis of various cell types (Beattie et al., 2000). Specifically, following SCI, damaged neurons release high concentrations of the neurotransmitter glutamate (Park et al., 2004) resulting in Ca^2+^ dysregulation, compromising cellular machinery (Herrero-Mendez et al., 2009), and increasing cell death (Duchen, 2012) along the apoptotic-necrotic continuum (Cheung et al., 1998).

Ca^2+^ entry into neurons and glia in the CNS is mediated by several channels, including voltage-gated Ca^2+^ channels (VGCCs; Sattler et al., 1996; Agrawal et al., 2000) ionotropic P2X_7_ receptors (Hollmann et al., 1991; North, 2002; Matute et al., 2007) and α-amino-3-hydroxy-5-methyl-4-isoxazolepropionic acid (AMPA) receptors lacking the GluR2 subunit (Hollmann et al., 1991). Excessive Ca^2+^ influx or Ca^2+^ -mediated intracellular Ca^2+^ release following an injury can activate several Ca^2+^-dependent processes leading to the overproduction of mitochondrial free radicals, and oxidative damage such as lipid peroxidation (Camello-Almaraz et al., 2006; O’Hare Doig et al., 2014). Lipid peroxidation occurs when reactive oxygen species react with polyunsaturated fatty acids, to form lipid radicals such as 2-propenal (acrolein) and 4-hydroxynonenal (HNE; Hamann and Shi, 2009; Vaishnav et al., 2010) consequently disturbing cellular machineries (Refsgaard et al., 2000). Free radical production and lipid peroxidation following SCI plays an important role in secondary injury (Hall and Braughler, 1982; Barut et al., 1993; Springer et al., 1997; Lewén et al., 2000; Kamencic et al., 2001; Christie et al., 2008).

Neuropathological changes in myelin architecture contribute to deficits in locomotor function following SCI (Guest et al., 2005) Changes seen in myelin structure may reflect disruption in the numbers of oligodendroglia along the differentiation lineage, resulting in perturbed axoglial support (Nave and Trapp, 2008). We have previously demonstrated increased nitrosative and oxidative damage in CC1^+ve^ mature myelinating oligodendrocytes *in vivo* following a partial injury to the optic nerve (O’Hare Doig et al., 2014) temporally and spatially associated with changes in the node of Ranvier and paranode axoglial structure (Szymanski et al., 2013). Oligodendrocytes and their progenitors (oligodendrocyte progenitor cells; OPCs) are reported to be particularly vulnerable to oxidative events (Giacci et al., 2018b), initiating necrotic and apoptotic pathways during CNS injury and neurodegenerative disorders such as multiple sclerosis (Bunge et al., 1993; Thorburne and Juurlink, 1996; Juurlink et al., 1998; Gilgun-Sherki et al., 2004; Jana and Pahan, 2007) associated with myelin abnormalities (Payne et al., 2012, 2013; Szymanski et al., 2013; Giacci et al., 2018a) demyelination (Griffiths et al., 1998; Matute et al., 2001; Antony et al., 2004; Norenberg et al., 2004; Doan et al., 2013) and delayed degeneration of axons (Crowe et al., 1997; Warden et al., 2001; Irvine and Blakemore, 2008).

Given the significant effects of excessive Ca^2+^ influx on cellular structure and function, there has been an increased effort in the use of Ca^2+^ (or ion) channel inhibitors as a treatment strategy for neurotrauma. Many of these agents such as Lomerizine (Lom), 2,3-dioxo-7-(1H-imidazole-1-yl)6-nitro-1,2,3,4-tetrahydro-1-quinoxalinyl acetic acid monohydrate (YM872), and adenosine 5′-triphosphate periodate oxidized sodium salt (oxATP) to inhibit VGCCs, Ca^2+^ permeable AMPA receptors, and P2X_7_ receptors, respectively, have been developed and tested in pre-clinical models and clinical trials of Neurotrauma. Taken together, although promising outcomes have been demonstrated in pre-clinical studies of Ca^2+^ channel inhibitors, clinical trials utilizing these agents alone for the treatment of neurotrauma alone have been limited and overall disappointing (for review see O’Hare Doig and Fitzgerald, 2015).

To facilitate functional recovery following CNS injury, and in particular SCI, therapeutic strategies must overcome the volatile environment, preserve neuronal and glial cell numbers, and limit changes in Ca^2+^ dynamics, oxidative stress, and myelin abnormalities. It is increasingly recognized that combinatorial treatment strategies are likely to be required to maximize limitation of the multiple detrimental facets of neurotrauma (Tuszynski, 2005; Kelso et al., 2011). Given the complexity of the pathophysiology of CNS trauma, we have previously assessed the efficacy of a variety of combinations of ion channel inhibitors: Lom, YM872, and OxATP both *in vitro* and *in vivo*. All three ion channel inhibitors in combination were shown to reduce intracellular Ca^2+^ concentration, increase cortical cell viability, and preserve astrocytes and neurons following peroxide insult *in vitro* (O’Hare Doig et al., 2016). Only application of Lom, YM872 and oxATP in combination *in vivo* up to 3 months following partial injury to the optic nerve limited chronic myelin decompaction and node of Ranvier abnormalities, associated with preservation of optokinetic reflex, indicating preservation of function, in this model; individual inhibitors were less effective (Savigni et al., 2013). Similarly, we have demonstrated the efficacy of this ion channel inhibitor combination in acute partial CNS injury, beneficial outcomes including reduced hyperphosphorylation of tau, acetylated tubulin, and lipid peroxidation; increased Nogo-A immunoreactivity, and preservation of AnkG lengths and OPC numbers (O’Hare Doig et al., 2017). Therefore, it is clear that a combination of ion channel inhibitors targeting different pathways is beneficial in dampening the biochemical sequelae and secondary cascade processes. However, the efficacy of the treatment strategy must be assessed in more clinically relevant models of neurotrauma such as SCI.

Therefore, this study was designed to further assess the efficacy of the ion channel inhibitor combination of Lom, oxATP, and YM872 on key events of secondary degeneration, during the acute and chronic time phases following SCI. The Infinite Horizon impactor device was utilized to provide a clinically relevant, and reproducible moderate thoracic contusion model of SCI in rodents (Anderson and Stokes, 1992), and to mimic the pathophysiology of SCI seen in humans.

## Materials and Methods

### Animals

Female Fischer rats (F344; 150–200 g) were bred at the Animal Resources Centre (ARC; Murdoch, WA, Australia), housed under a standard 12 h light/dark cycle, fed wet and dry rat chow and water *ad libitum*. All procedures performed complied with the ARRIVE guidelines and were carried out following the “Guide for the Care and Use of Laboratory Animals (National Institutes of Health Publication No. 8023, revised 1978), and were approved by The University of Western Australia’s Animal Ethics Committee (AEC, approval number RA/3/100/1405). Animals were of adult age at the time of experimental testing (12–15 weeks old).

### Treatments

Animals (*N* = 28) were randomly allocated into two cohorts: Cohort 1 (2 Weeks; 2W); SCI plus PBS vehicle (2W control; *n* = 7) or SCI plus ion channel inhibitors (2W treated; *n* = 7); Cohort 2 (10 Weeks; 10W), SCI plus PBS vehicle (10W control; *n* = 7), or SCI plus ion channel inhibitors (10W treated; *n* = 7). Treated animals were administered all three ion channel inhibitors in combination: Lom (LKT Labs, St. Paul, MN, USA), oxATP (Sigma, St. Louis, MO, USA), and YM872 (LKT Labs, St. Paul, MN, USA). The choice of treatment concentrations, length, and timing of delivery was based on previously published studies demonstrating efficacy using these agents individually and demonstration of efficacy in the partial optic nerve transection model (Fitzgerald et al., 2009a,b; Savigni et al., 2013). Therefore, Lom and butter vehicle alone treatments began on the day of surgery, after recovery from anesthesia and continued twice daily for 2 or 10 weeks (cohort dependent). Lom mixed in butter vehicle (30 mg/kg body weight; Tamaki et al., 2003) or butter vehicle alone was administered orally to all animals using a spatula, whilst animals were gently held. OxATP (1 mM; Matute et al., 2007) and YM872 (240 μM; Savigni et al., 2013) were delivered directly to the site of injury for the first 2 weeks after injury *via* an osmotic mini-pump (see below).

### Anesthesia and Surgery

Adult Fisher rats were anesthetized with 2.5% (v/v) isoflourothane (BoMac Attane Isoflurane, Hillcrest, Auckland, NZ, USA) combined with 60% nitrous oxide (v/v) and 38% oxygen (v/v) for a quick and controlled recovery. A longitudinal incision through the skin and spreading of the underlying muscle tissue was performed, revealing the spinal column. Rats were positioned on a surgical plate for spinal cord impact using flexible armatures and Adson forceps (spinal cord stabilizing forceps). Partial laminectomy at vertebral level T9-T11 exposed the spinal cord underneath without disruption of the meninges. Using an Infinite Horizon impactor device, animals received a moderate T10 contusion (150 kDyne) injury to the dorsal surface of the exposed spinal cord. Following contusion injury, the dura was incised longitudinally, and a pre-filled osmotic mini-pump (Model 2002; 0.5 μl/h; ALZET, Cupertino, CA, USA) attached to a brain infusion kit (Kit 3; ALZET, Cupertino, CA, USA) were attached and stabilized *via* subcutaneous sutures. The vinyl catheter was guided underneath the skin, the brain infusion cannula was placed above the dorsal aspect of the exposed spinal cord, and the 30-gauge stainless steel tube was inserted directly into the injury site. The cannula was sutured in place, muscles were closed in layers and the incision closed with wound clips. Rehydrating saline (2 ml subcutaneously) was administered in conjunction with Buprenorphine (Temgesic; 0.01 ml/100 g body weight, [(0.0324 mg/kg body weight), 300 U/ml; Provet, Malaga, WA, Australia] immediately following surgery, and daily until ~day 5 post-SCI. To prevent wound and bladder infections, Benacillin [0.02 ml/100 g body weight (64 mg/kg body weight), 300 U/ml (150 mg/ml procaine penicillin, 150 mg/ml benzathine penicillin, 20 mg/ml procaine hydrochloride); Troy Laboratories Pty. Ltd., Glendenning, NSW, Australia] was administered immediately after and at 2-, 4- and 6-days following surgery. The ALZET brain infusion kits and pumps only remained *in situ* for 2 weeks following SCI, as previous studies have demonstrated reduced efficiency in delivery, and sometimes, moderate compression damage caused by tubing, at later time points (Jones and Tuszynski, 2001; Hodgetts et al., 2013). Therefore, pumps were surgically removed under anesthesia (as described above) at 2 weeks after injury.

### Behavioral Analyses—Open Field Recovery (BBB)

Functional assessments of animals in all experimental groups were performed on days 1–7, then weekly from weeks 2–10 following surgery (cohort dependent) and consisted of open field locomotion assessment. Functional assessment was conducted before surgical removal of osmotic mini-pumps at the 2-week time-point for Cohort 2 animals. Handling and habituation of animals to behavioral apparatus were performed during the week before surgery.

The Basso, Beattie and Bresnahan (BBB) locomotor rating scores were used to assess the range and type of forwarding locomotion (Basso et al., 1995). Briefly, rats were recorded using digital videography in an open field for 2–3 min (per hindlimb) on days 1–7, and weekly from week 2–10 post-injury. Recordings were made from a sideways angle, at close range for each hindlimb, as well as views from behind the animals during the test. Scoring of animals ranged from 0 to 21 based on the criteria outline (Basso et al., 1995). At least two independent investigators conducted the assessments, all blinded to experimental group identity. Both scores for each hindlimb were averaged, and the total average score was reported for each animal.

### Tissue Preparation

**Figure 1:**
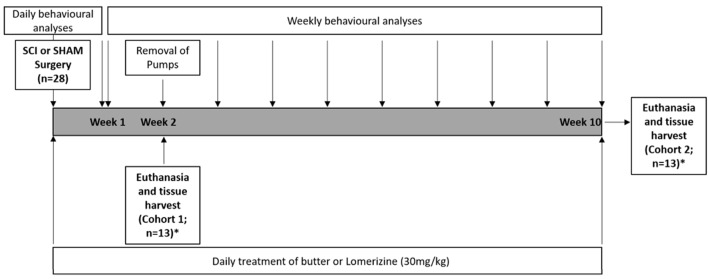
Study design. Following sham or spinal cord injury (SCI) surgery, two cohorts of animals (*N* = 28) were treated daily with butter (orally) or lomerizine (30 mg/kg, orally), and saline or oxATP + YM872 *via* osmotic mini-pump. Daily behavioral analyses were conducted for the first week following surgery, and then weekly thereafter. Surgical removal of pumps occurred in Cohort 2 animals, only. At 2 weeks (Cohort 1; *n* = 14) or 10 weeks following surgery (Cohort 2; *n* = 14), animals were humanely killed and tissue was harvested for analysis. *Total number of animals included in behavioral and histological analysis.

Following final behavioral tests at 2- or 10-weeks post-injury, animals were euthanized by lethal injection of sodium pentobarbitone (50 mg/100 g; Provet, Malaga, WA, Australia) and transcardially perfused with heparinized PBS, followed by 4% (w/v) paraformaldehyde (PFA; Sigma) in 0.1 M phosphate buffer (pH 7.2). The vertebral columns were dissected from each animal and postfixed in PFA for 24 h before cryoprotection in 15% (w/v) sucrose in phosphate-buffered saline (PBS; pH 7.2). A 20 mm segment was cut from the spinal cord (containing the lesion epicenter and rostral-caudal penumbra), ensuring the injury site was at the midpoint, and embedded in 10% (w/v) gelatine (Sigma, St. Louis, MO, USA) in PBS. Gelatine blocks were trimmed and frozen at −20°C, then embedded in optimal cutting temperature (OCT) compound (ProSciTech; Kirwan, QLD, Australia) and a cryostat (CM1900, Leica; Wetzlar, Germany) used to cut longitudinal spinal cord sections (35 μm). Consecutive series of sections were transferred to 24-well plates containing PBS + 0.1% (w/v) sodium azide (Sigma, St. Louis, MO, USA) and stored at 4°C until processed for immunohistochemistry (Figure 1).

### Cyst Size Analysis

Quantification of cyst size and tissue sparing was performed by analysis of toluidine blue-stained spinal cord sections. Every seventh longitudinal section of the spinal cord was mounted on slides and air-dried overnight. For all animals, the selected sections were from the same relative location, confirmed by histological analysis. Sections were submerged in 0.05% (w/v) toluidine blue [in 0.0055% (w/v) sodium tetraborate; Sigma, St. Louis, MO, USA] for 1 min then rinsed and dehydrated sequentially in 70%, 90% and 100% (v/v) ethanol for 3 min each. Slides were then submerged in xylene for 1 min, cover-slipped using DPX slide mounting medium (Sigma, St. Louis, MO, USA), and then stored at room temperature before imaging. Data from each of the stained sections were combined and analyzed to give a total cyst area measurement, and total tissue area measurement, for each animal. The cyst area was defined as the outline of the cyst within the spinal cord section, and tissue area was defined as the outline of the entire spinal cord section within each field of view (FOV), using Fiji image analysis software (Schindelin et al., 2012). The total area of cystic structures was expressed as a percentage of total tissue area per FOV. Whilst there was the possibility of cyst generation caused by catheter placement in the spinal cord parenchyma, the track of the catheter (if any) in spared tissue was visible in sections. As they are typically very thin and distinctly different from the underlying cyst, they were identified (if any) and excluded from the analysis.

### Immunohistochemical Assessment

Selected sections within the lesion epicentre were washed in PBS and immunohistochemical analyses conducted according to established procedures (Fitzgerald et al., 2010; Hodgetts et al., 2013) using primary antibodies recognising: astrocytes, glial fibrillary acid protein (GFAP; 1:1,000; #ab53554, Abcam, Cambridge, UK); neurons, β-III-tubulin (1:1,000; MMS-435P-250, Jomar Bioscience, Scoresby, VIC, Australia); non-phosphorylated neurofilaments of mature motor neurons (SMI-32; 1:1,000; #NE1023, Merck Millipore, Burlington, MA, USA); growth associated protein 43 (GAP43; 1:500; #SAB4300525, Sigma, St. Louis, MO, USA); myelin basic protein (MBP; 1:500; #ab40390, Abcam, Cambridge, UK); mature oligodendrocytes (CC1; 1:500; #ab16794, Merck Millipore, Burlington, MA, USA); oligodendrocyte progenitor cells (OPCs); platelet-derived growth factor alpha (PDGFα; 1:500; #sc-9974, Santa Cruz Biotechnology, Dallas, TX, USA) and neural/glial antigen 2 (NG2; 1:500; #ab83178, Abcam, Cambridge, UK); lipid peroxidation markers, acrolein (1:500; #ab37110, Abcam, Cambridge, UK); 4-Hydroxynonenal (HNE; 1:500; #STA-035, Jomar Bioscience, Scoresby, VIC, Australia), and Hoechst nuclear stain (1:3,000; #62249, Invitrogen, Carlsbad, CA, USA). Secondary antibodies were species-specific AlexaFluor^®^ 488, 555, and 647 (1:400; Life Technologies, Carlsbad, CA, USA) respective for each primary antibody.

### Immunohistochemical Analysis and Semi-quantification

**Figure 2:**
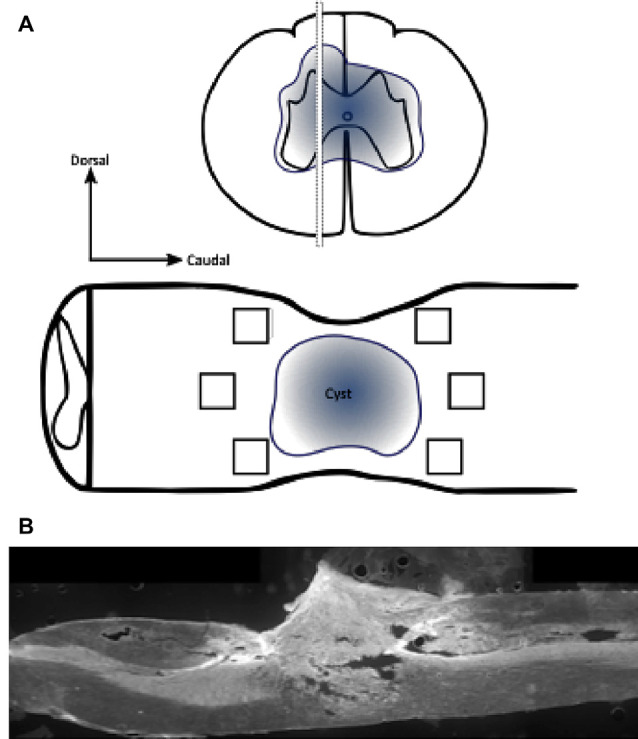
Schematic diagram demonstrating regions of interest (ROIs) in a selected section of the spinal cord for immunohistochemistry. Immunohistochemical labeling was visualized in a selected section of the spinal cord, in the immediate penumbra surrounding the lesion site (cyst). Images were collected from six 200 μm × 200 μm regions of interest (black box; dorsorostral, centrorostral, ventrorostral, dorsocaudal, centrocaudal, and ventrocaudal) immediately adjacent to the injury site (**A**; not to scale). Stitched ×10 gray scale fluorescent image demonstrates cord injury site and evidence of cystic structures **(B)**.

Immunohistochemical labeling was visualized in a single selected section of the spinal cord, in the immediate area surrounding the lesion site for each animal and photographed using either an upright Eclipse E800 microscope (Nikon Corporation, Minato, Tokyo, Japan) or, where co-localization and cellular densities were quantified, a Nikon Eclipse Ti inverted microscope (Nikon Corporation, Minato, Tokyo, Japan). For all outcomes and tissues, images were collected at set constant exposures in a single sitting where possible, and immunointensity analyses were conducted on six 200 μm × 200 μm regions of interest (ROIs; dorsorostral, centrorostral, ventrorostral, dorsocaudal, centrocaudal and ventrocaudal) immediately adjacent to the injury site (Figure 2) using Fiji analysis software (Schindelin et al., 2012). Constant arbitrary threshold intensities for all images for a marker relative to the background (black, no tissue) were set to account for potential variation in section thickness and antibody application, and mean intensities and areas above-set threshold were semi-quantified. As normalization to the background is not possible for oxidative stress data, as oxidized proteins and DNA are diffusely distributed throughout individual cells and or tissue, data relative to other proteins was not normalized. Note that pilot analyses comparing outcomes between the six ROI for analyzed tissue sections within each experimental group did not reveal differences and so data for the six ROI for each tissue section (i.e., animal) were averaged and these means used for further statistical interpretation. Only the mean intensity above threshold rather than the area of immunointensities above the threshold is shown in the following results unless stated within the text. Cell-type-specific densities were quantified through a series of optical images at six individual ROIs at 1 μm increments along the z-axis, acquired from the middle 9 μm of each cord section. All images were taken in areas surrounding the injury site, where no evidence of cystic structures was present. This ensured any observable changes in immunopositive areas and cell population densities were not due to physical disturbances of spinal cord tissue. Secondary antibody-only controls were processed concurrently to ensure the selectivity of staining (data not shown).

### Statistical Analyses

Mann–Whitney *U* test for non-parametric data was used to analyze open field locomotion testing (BBB analysis) data, comparing control vs. treated at each time point in separate analyses for the animals euthanized at 2 or 10 weeks. Statistical analyses of cyst size and semi-quantitative immunohistochemical assessments were conducted using a two-way ANOVA followed by Sidak’s multiple comparisons test. All statistical analyses were performed using GraphPad Prism version 7.00 (GraphPad Software). All data assumes equal variance unless otherwise stated. All data were presented as the mean ± standard error of the mean. Adjusted *P* values ≤ 0.05 were considered statistically significant. For *post hoc* analyses of cyst size and immunohistochemical assessments, comparisons were made between 2W control vs. 2W treated, 10W control vs. 10W treated, 2W control vs. 10W control, and 2W treated vs. 10W treated. No sample calculation was performed. The sample size was based on previous research in our laboratory.

## Results

### Short Term Combinatorial Ion Channel Inhibitor Treatment Facilitated Significant Early Hindlimb Functional Recovery That Was Not Sustained

**Figure 3:**
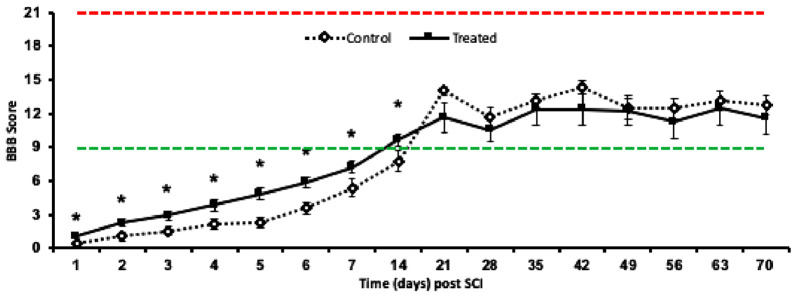
Mean ± SEM locomotor function assessment (BBB) scores in the open field recovery assessment of hindlimb motor function, up to 10 weeks post-SCI. Animals treated with Lom, oxATP, and YM872 (dashed black line) compared to vehicle-treated control animals (solid black line; *p ≤ 0.05). Red dashed line represents a typical score of completely uninjured, normal animals (BBB = 21) and the green dashed line represents weight supporting BBB score (BBB = 9). No significant differences were observed between 2W and 10W cohorts (data not shown), therefore only 10W cohort shown (*n* = 6–7; *n* = animal numbers).

Following injury, animals demonstrated significant locomotor deficits in the hind limbs. Animals demonstrating weight support on the hindlimbs (BBB score ≥9) 24 h following SCI was excluded from both behavioral and histological analysis. These observations typically correlate with an unsuccessful impact of the cord. This only occurred once in each cohort, of the vehicle-treated groups. One animal from 2W + PBS and 10W + PBS groups were removed from the study analysis. Two cohorts of rats were scored daily for 7 days, and then once weekly thereafter on their ability to generate spontaneous, forward movement, from the day of surgery until endpoint: 14 days (2W: *acute*) or 70 days (10W: *chronic*) following SCI. During the 2 weeks of continuous combinatorial administration of ion channel inhibitors, treated animals (of Cohort 1 and 2) demonstrated significant improvement in locomotor function compared to control as early as 1 day and sustained until up to 14 days post-SCI (*p* ≤ 0.05). No significant differences in locomotor movements were observed between control and treated groups from 21 days to 70 days post-SCI (Cohort 2; *p* ≤ 0.05; Figure 3; Cohort 2 data shown).

### Ion Channel Inhibitor Treatment Reduced Cyst Size and Acute Reactive Gliosis Following SCI

**Figure 4:**
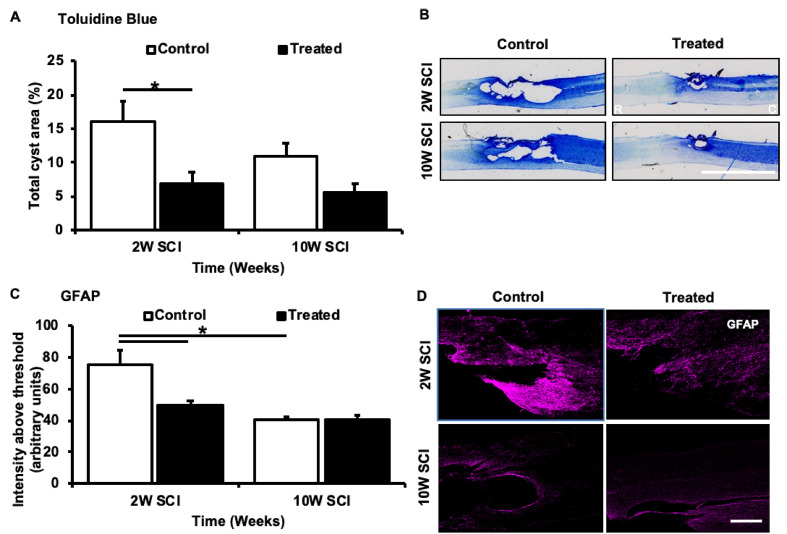
Effects of Lom, oxATP, and YM872 on cyst formation and reactive gliosis of GFAP^+ve^ astrocytes, at 2W and 10W post-SCI. **(A)** Mean ± SEM of cyst area in toluidine blue-stained spinal cord sections (*n* = 6–7; *p ≤ 0.05). **(B)** Representative images of rostral (R) and caudal (C) regions of a single spinal cord section (scale bar = 5 mm). **(C)** Mean ± SEM intensity above the threshold of GFAP immunoreactivity (*n* = 6–7; *p ≤ 0.05). **(D)** Representative images of areas GFAP^+ve^ (magenta) immunoreactivity surrounding the injury site (scale bar = 500 μm; *n* = animal numbers).

Cyst formation was calculated as a percentage of the damaged area relative to spared tissue, within each spinal cord segment, 2- and 10-weeks following SCI. Treatment with ion channel inhibitors significantly reduced the cyst area, compared to the relative control groups at 2W (*F*_(1,22)_ = 3.126, *p* = 0.0303). However, there was no significant reduction in cyst size following treatment at 10W (*F*_(1,22)_ = 1.73, *p* = 0.462). Similarly, cyst size did not change across time for either control (*F*_(1,22)_ = 0.153, *p* = 0.460) or treated animals (*p* = 0.725; Figures 4A,B). To determine whether the combination of ion channel inhibitors modified the inhibitory environment surrounding the injury site, the effects of ion channel inhibitors on reactive astrogliosis were assessed. Semi-quantification of GFAP immunoreactivity demonstrated that treated animals at 2 weeks post-SCI had significantly reduced intensity above threshold compared to 2-week controls (*F*_(1,22)_ = 3.677, *p* = 0.009). However, no effects of treatment were observed between treated and control animals at 10 weeks post-SCI (*F*_(1,22)_ = 0.028, *p* = 0.734). Although a significant difference was observed between 2W control and 10W control animals (*F*_(1,22)_ = 4.629, *p* = 0.001), there was no significant difference between 2W treated and 10W treated animals (*F*_(1,22)_ = 1.330, *p* = 0.7347; Figures 4C,D).

### Ion Channel Inhibitor Treatment Promoted Changes in βIII-Tubulin Immunoreactivity, but Not Regenerative Capacity, Within Spared Axons During Acute SCI

**Figure 5:**
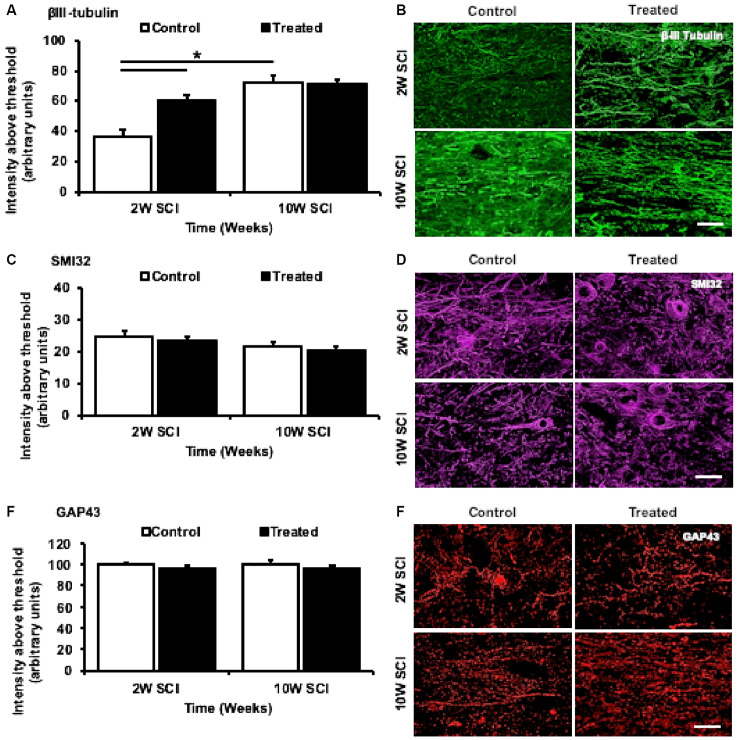
Effects of Lom, oxATP, and YM872 on βIII-tubulin, SMI32, and GAP43, at 2W and 10W post-SCI. **(A–F)** Mean ± SEM of βIII-tubulin **(A)**, SMI32 **(C)**, and GAP43 **(E)**, immunointensity above threshold (*n* = 6–7; *p ≤ 0.05). Representative images show areas of βIII-tubulin^+ve^ (green; **B**), SMI32^+ve^ (magenta; **D**) and GAP43^+ve^ (red; **F**) immunoreactivity surrounding the injury site (scale bars = 50 μm; *n* = animal numbers).

To determine whether the combinatorial ion channel inhibitor therapy preserved axonal integrity and/or promoted axonal sprouting/regeneration following SCI, βIII-tubulin (Roskams et al., 1998) SMI32 (Carriedo et al., 1996), and GAP43 (Aigner et al., 1995) immunoreactivity were semi-quantified. Following SCI, 2W treated animals had a significant increase in βIII-tubulin intensity above threshold compared to 2W controls (*F*_(1,22)_ = 4.279, *p* = 0.002). This effect was not observed in 10W treated animals (*F*_(1,22)_ = 0.292, *p* > 0.999). Again, there was a significant difference between 2W control and 10W control animals (*F*_(1,22)_ = 6.277, *p* < 0.001), however the same difference was not observed between 2W treated and 10W treated animals (*F*_(1,22)_ = 2.022, *p* = 0.290; Figures 5A,B). To further determine whether this significant effect was due to sparing of βIII-tubulin positive axons, the area above threshold, indicative of the number of axons within an area (each ROI), was also analyzed. However, no significant changes were observed between treated or control animals (data not shown). Therefore, it is unlikely that the inhibitors spared axons, but instead there were significant increases in tubulin immunoreactivity within spared axons, perhaps due to conformational alterations. There were no significant differences observed between groups in intensity or area above threshold of SMI32 immunoreactivity at 2W, or 10W following SCI (*F*_(1,22)_ = 0.685, *p* = 0.0.990; Figures 5C,D). Similarly, there were no significant effects of treatment (*F*_(1,22)_ = 0.300, *p* = 0.589) or time (*F*_(1,22)_ = 2.596, *p* = 0.121) between animals in GAP43 immunointensity, 2W or 10W following SCI, suggesting no effect of injury or treatment on axonal regeneration (Figures 5E,F).

### Preservation of MBP Associated With Density Changes in CC1^+ve^/NG^−ve^ and NG2^+ve^/PDGFα^+ve^ Oligodendroglia During Acute SCI

**Figure 6:**
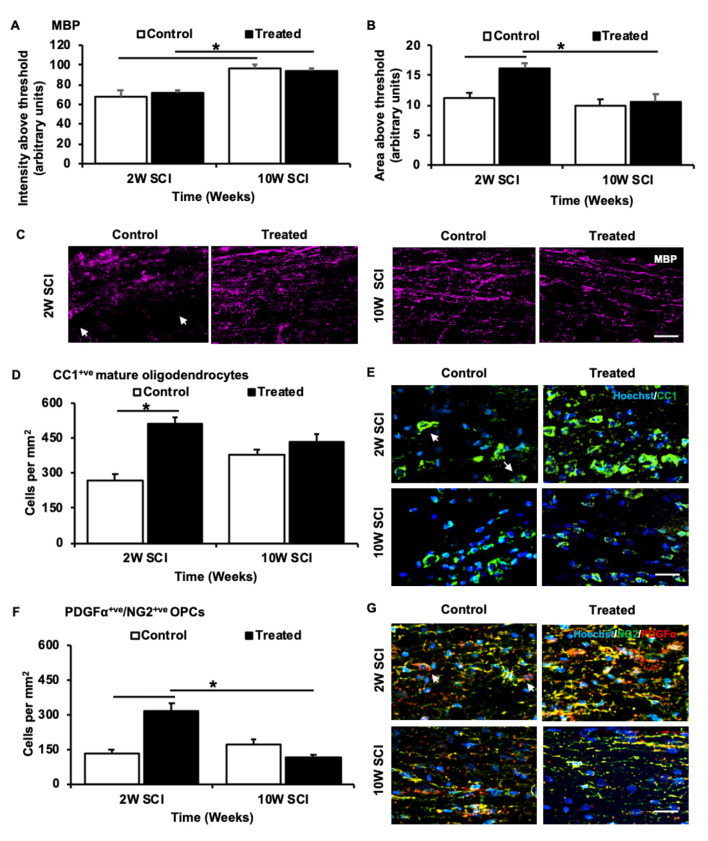
Effects of Lom, oxATP, and YM872 on myelin basic protein (MBP) and oligodendroglia sub-populations, at 2W and 10W post-SCI. **(A)** Mean ± SEM intensity above the threshold, and **(B)** mean ± SEM area above the threshold of MBP (*n* = 6–7; *p ≤ 0.05). **(C)** Representative images show areas of MBP^+ve^ (magenta) immunoreactivity surrounding the injury site. White arrows demonstrate large area regions where MBP was not detected (scale = 50 μm). **(D)** Mean ± SEM mature oligodendrocytes cells per mm ^2^ (*n* = 6–7; *p ≤ 0.05). **(E)** Representative images show the density of CC1^+ve^ NG2^−ve^ mature oligodendrocytes (green; indicated by white arrows) surrounding the injury site (scale = 25 μm). **(F)** Mean ± SEM OPCs per mm^2^ (*n* = 6–7; *p ≤ 0.05). **(G)** Representative images show the density of NG2^+ve^/PDGFα^+ve^ OPCs (green/red; indicated by white arrows) surrounding the injury site (scale = 25 μm; *n* = animal numbers).

To determine the effects of combinatorial ion channel inhibitor therapy on myelin, oligodendrocytes and their progenitors following SCI, MBP immunoreactivity was assessed, and the densities of CC1^+ve^/NG2^−ve^ mature oligodendrocytes and NG2^+ve^/PDGFα^+ve^ OPCs were quantified. Following treatment, 2W animals had no significant changes in MBP immunointensity (*F*_(1,22)_ = 0.66, *p* = 0.987; Figure 6A), but instead were observed to have a significant increase in MBP immunopositive area above the threshold, when compared to 2W control animals (*F*_(1,22)_ = 3.057, *p* = 0.0036; Figure 6B). There was an effect of time, with a significant difference observed between the MBP immunointensity observed in 2W control and 10W treated animals (*F*_(1,22)_ = 4.101, *p* = 0.003). However, no significant differences in MBP intensity or area above the threshold were observed between 10W treated and control animals (*F*_(1,22)_ = 0.013, *p* < 0.999; Figures 6A–C). Quantification of CC1^+ve^/NG2^−ve^ cells indicated a significant increase in mature oligodendrocyte numbers in treated animals compared to 2W controls (*F*_(1,22)_ = 4.879, *p* < 0.001). However, there were no significant differences between 2W and 10W control (*F*_(1,22)_ = 2.102, *p* = 0.250) or 2W and 10W treated animals (*F*_(1,22)_ = 1.72, *p* = 0.462), and there were no significant differences in the density of mature oligodendrocytes at 10W when comparing treated animals and controls (*F*_(1,22)_ = 0.930, *p* = 0. 9327; Figures 6D,E). Quantification of NG2^+ve^/PDGFα^+ve^ cells indicated the density of OPCs was significantly increased at 2W, following treatment with ion channel inhibitors (*F*_(1,22)_ = 5.407, *p* < 0.001). But, at 10W following SCI, the significant effect of treatment was no longer present (*F*_(1,22)_ = 2.67, *p* = 0.085). Also, there was a significant reduction in OPCs when comparing 10W treated to 2W treated animals (*F*_(1,22)_ = 6.610m *p* < 0.001), but no difference between 2W and 10W controls (*F*_(1,22)_ = 1.891, *p* = 0.366; Figures 6F,G).

### Ion Channel Inhibitor Treatment Limited Lipid Peroxidation During Early SCI

**Figure 7:**
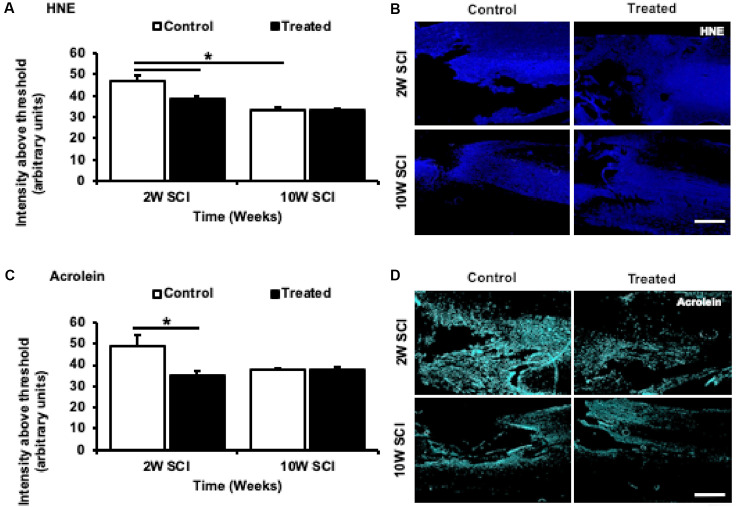
Effects of Lom, oxATP, and YM872 on lipid peroxidation by-products, 4-hydroxynonenal (HNE), and acrolein, at 2W and 10W post-SCI. **(A–D)** Mean ± SEM intensity above the threshold of lipid peroxidation by-products 4-hydroxynonenal (HNE) **(A)** and acrolein (**C**; *n* = 6–7; *p ≤ 0.05). Representative images show areas of HNE^+ve^ (blue; **B**) and acrolein^+ve^ (cyan; **D**) immunoreactivity surrounding the injury site (scale bars = 500 μm; *n* = animal numbers).

The effects of combinatorial ion channel inhibitor therapy on oxidative by-products caused by lipid peroxidation were also assessed. Immunohistochemical analysis of lipid peroxidation by-products revealed 2W treated animals had significant reductions in both HNE (*F*_(1,22)_ = 4.090, *p* = 0.0034) and Acrolein (*F*_(1,22)_ = 3.677, *p* = 0.008) immunoreactivity compared to 2W control animals. Although there was a significant reduction in HNE immunoreactivity with time when comparing 2W and 10W control animals (*F*_(1,22)_ = 6.134, *p* < 0.001), there were no significant effects of treatment on HNE (*F*_(1,22)_ < 0.001, *p* > 0.999) or Acrolein (*F*_(1,22)_ = 0.003, *p* > 0.999) immunoreactivity at 10W following SCI (Figures 7A–D).

## Discussion

The current study was designed to assess the efficacy of a combination of ion channel inhibitors; Lom, oxATP, and YM872, in a clinically relevant rodent model of the contusion-injured spinal cord. This combination of inhibitors has been previously assessed in well established, high throughput *in vitro* and *in vivo* models of acute and chronic CNS injury, consistently demonstrating significant beneficial effects on Ca^2+^ influx, cell death, oxidative stress, myelin and node of Ranvier changes, as well as visual function (Savigni et al., 2013; O’Hare Doig et al., 2014). This study aimed to assess the effects of the ion channel inhibitor combination on key events of CNS injury, during the acute (2 weeks) and chronic time phases (10 weeks) following SCI. Following treatment with ion channel inhibitors, there was a significant increase in acute functional hindlimb recovery and reduced formation of cystic structures and reactive gliosis surrounding the injury site. These changes were associated with acute increases in βIII-tubulin and myelin protein immunoreactivity, as well as significant effects on oligodendrocytes and their progenitors, and significant reductions in lipid peroxidation products, acrolein, and HNE. However, all observed effects of treatment (observed acutely) were not seen in chronic SCI animals, once delivery of oxATP and YM872 was removed.

In this study, treatment with Lom, YM872, and oxATP together caused a significant increase in the intensity of βIII-tubulin^+ve^ profiles surrounding the injury site, in the thoracic spinal cord. This was associated with reduced formation of cystic structures, as well as improved functional recovery. However, following 10-week treatment, despite an acute reduction of cyst size (increased tissue-sparing), the immunointensity of β-III tubulin staining, and animal locomotor recovery were no longer significantly different from vehicle control. This suggests the treatment effect is only in the first 2 weeks following SCI when all inhibitors are present. β-III tubulin is a protein associated with dynamic microtubule extension in neurons (Lee et al., 1990) and although not neuronal-specific, is a useful indicator of re-growing axons (Avwenagha et al., 2003). However, there was no significant increase in GAP43, a marker expressed in elevated levels during development and regeneration, and shown to be up-regulated in growing axons following compressive SCI (Curtis et al., 1993). Thus, it is unlikely that the combination of ion channel inhibitors promoted the regeneration of axons, or altered the endogenous regenerative capacity of these neurons. Instead, the treatment resulted in acute changes in βIII-tubulin profiles within spared axons, independent of GAP43 upregulation. This may be due to increased (at least temporally) microtubule stabilization, known to reduce scarring and promote axon regeneration after SCI (Hellal et al., 2011). This is plausible, given the increase in βIII-tubulin profiles and a decrease in cyst area seen in untreated SCI animals, temporally (2W control vs. 10W control animals). Interestingly, no significant effects of treatment were observed in SMI32^+ve^ profiles (large, mature motor neurons). This may further highlight a selective vulnerability to damage and/or stress of different sub populations of neurons, as shown previously (Morrison et al., 1996; Saroff et al., 2000; Greene et al., 2005; Ren et al., 2005; Wang and Michaelis, 2010). Compared to bipedal primates, quadrupedal mammals typically have a much greater reliance on sensory input from limbs to drive load-bearing spinal locomotor movements. Therefore, rodents typically are much more capable of supported hindlimb stepping in the absence of supraspinal input (Côté et al., 2017). Given the final BBB scores of the current study are in the range of 12–14 (chronic animals), we speculate that there is likely little preservation of supraspinal control driving hindlimb functional recovery. As such, final BBB scores are not entirely reflective of the persistent reduction in cyst size (tissue preservation) that was achieved with combinatorial treatment at 2- and 10-weeks post-injury. Inclusion of other functional tests such as the Louisville Swim Scale (Smith et al., 2006) where tactile inputs from fore- and hindlimb are diminished could confirm the extent of supraspinal damage. In conjunction, future studies could combine dye tracing antero- and retrograde dye tracing techniques with an assessment of the density of subpopulations of neurons and their axonal projections, utilizing additional neuronal markers including, but not limited to: calcitonin gene-related peptide to assess sensory neurons (Wiesenfeld-Hallin et al., 1984) and tryptophan, and tyrosine hydroxylase to assess dopaminergic and serotonergic neurons, respectively. Changes in the population of individual subpopulations of cells may reflect specific effects of ion channel inhibitors on particular neuronal subtypes (Van Den Bosch et al., 2000), and may provide greater insight and additional utility for the treatment of SCI. While the effects of the inhibitor combination on axons are not yet fully elucidated, there is a clear association observed between myelin, oligodendrocyte populations, and lipid peroxidation surrounding the injury site.

We have previously demonstrated a host of pathophysiological events that occur during secondary degeneration, including the formation of free radicals, associated with lipid peroxidation (O’Hare Doig et al., 2014). HNE has been shown to rapidly accumulate and consequently inhibit glutamate uptake, following SCI, causing neuronal dysfunction and apoptosis (Springer et al., 1997). Similarly, protein-bound acrolein has also been observed to accumulate, up to 7 days following compressive SCI (Luo et al., 2005). In a recent acute study of partial optic nerve injury, we showed that treatment with Lom, oxATP, and YM872 in combination, reduced various elements of oxidative stress, including lipid peroxidation (O’Hare Doig et al., 2017) associated with preservation of OPCs. In the current study, the acute effects of these inhibitors on by-products of lipid peroxidation were associated with acute changes to MBP immunoreactivity in the injured spinal cord, at 2 weeks, but not 10 weeks following SCI. Similarly, the temporal reduction of HNE was associated with an increase in myelin profiles seen in non-treated control animals (2W vs. 10W). Oligodendrocytes are known to be vulnerable to oxidative damage following neurotrauma, particularly during proliferative phases and the genesis of myelin sheaths (Juurlink et al., 1998; Roth and Nunez, 2016; Giacci et al., 2018b). ROS, hypochlorous acid, and hydrogen peroxide, along with reactive nitrogen species, hydroxyl radicals and peroxynitrite are likely contributors to the destruction of oligodendroglia following CNS injury (O’Hare Doig et al., 2014). The current study demonstrated a significant increase in the density of OPCs and mature oligodendrocytes following a 2-week treatment with inhibitors, associated with reduced lipid oxidation. Interestingly, however, at 10 weeks, there was no significant reduction in the number of OPCs observed in treated animals, compared to 10-week control animals, associated with a lack of change in HNE and Acrolein. It has been established previously that the differentiation of OPCs into mature oligodendrocytes occurs in response to demyelination (Fancy et al., 2004). Therefore, the increased density of OPCs observed in 2 weeks treated animals may be a response to changes in myelin proteins (e.g., MBP), indicated by changes in MBP immunopositive areas. The increased number of CC1^+ve^ acutely may reflect the early survival of OPCs, which then differentiate into mature oligodendrocytes. Previous studies have demonstrated changes in proliferation and migration of the oligodendrocyte and OPC populations in response to demyelination following SCI (Keirstead et al., 1998; Totoiu and Keirstead, 2005). However, it is as yet unclear whether the observed changes in mature oligodendrocytes are the cause of MBP changes in the present study, in turn driving the accumulation and then differentiation of OPCs (Sim et al., 2002) Alternatively, an acute selective vulnerability of oligodendroglia to oxidative damage may be the underlying cause of myelin changes, and observed density changes of oligodendrocytes, and their progenitors, following SCI treatment (Back et al., 1998, 2001; Giacci et al., 2018b). Specifically, delayed oxidative damage in the sub-population of oligodendroglia may account for the decrease in numbers of OPCs observed in treated animals at 10 weeks following SCI, compared to 2 weeks. However, this interpretation remains a matter of speculation. Therefore, future assessment of myelin status using established electron microscopy measures (i.e., ratio) are warranted.

This is the first study to assess the effects of combined Lom, oxATP, and YM872 treatment in a clinically relevant model of SCI and provides early indications of efficacy of acute treatment. In this initial study, we have demonstrated efficacy with 2 weeks of treatment, however, further work will be needed in future studies to distinguish between the lack of sustained treatment or lack of efficacy to explain the 10-week outcome. Like many injury and disease state models, differences in treatment delivery, anatomy, physiology and relative scale between humans and other mammals leads to difficulties in interpretation. Rodent models of SCI provide a plethora of advantages compared to non-human primate models, with several reproducible methods well described, including established behavioral assessments (for an extensive review see Hodgetts et al., 2009). Several studies have shown the successful treatment of CNS injury using osmotic mini-pumps in rodents. However, many of these studies do not demonstrate efficacy following chronic implantation in the spinal cord, either due to experimental design (only measuring acute time points), complications exacerbated by the implantation of the pump, such as tissue ablation, catheter failure, dislodgment or obstruction, and inconsistent/adverse drug delivery (de Yebenes et al., 1987; Bear et al., 1990; Lu and Hagg, 1997; Angel et al., 1998; Hodgetts et al., 2013). These limitations demonstrate the difficulty of microinfusing blood-brain-barrier impermeable agents which must be given directly to the spinal cord and/or injury site. Despite such difficulties, we have demonstrated that the combined delivery of Lom, oxATP, and YM872 is associated with acute beneficial effects following SCI. However, chronic administration of oxATP and/or YM872 is likely needed to further improve the changes, such as functional improvements, seen at 2 weeks. Due to experimental design, we do not know if surgical procedures involving the removal of osmotic mini-pumps administrating these reagents, exacerbated further secondary events following SCI that were not measured in the current study, potentially hindering further functional improvements that may have resulted from the acute treatment. A literature review indicates that the longer-term effects of surgically removing osmotic mini-pumps have not been examined in depth (Jones and Tuszynski, 2001) and need to be explored further. Similarly, future studies should incorporate plasma/serum sampling to ensure the longitudinal functionality of the osmotic mini-pumps and inhibitor stability. Similarly, for clinical use, it will be necessary for future studies to determine how long after injury the combined treatment can be delayed, and efficacy maintained. In our optic nerve transection model, delayed combinatorial treatment by 6 h resulted in improvements in visual function, and the associated reduction in oxidative stress indicators (Yates et al., 2017). This suggests, time-delayed treatment (clinically relevant to acute SCI) of ion channel inhibitors remains a therapeutically viable option. In contrast, it might be that the ion channel inhibitors slow the rate of tissue loss, but ultimately not the final amount which may be due to impaired blood flow. This could be exploited as a way of buying time at the scene of emergency whilst patients are transported for more definitive treatments (e.g., decompressive surgery), and could be assessed in the acute phase (≤72 h) in pre-clinical studies.

Regardless, the current study was designed to assess the particular combination of inhibitors shown to be efficacious following partial optic nerve transection, in a clinically relevant model of SCI. However, to overcome the difficulties of microinfusion, blood-brain-barrier permeable drug alternatives are worth pursuing. Peng et al. (2009) found that an analog FD&C blue dye, No. 1, Brilliant Blue G (BBG), can be used to selectively antagonize P2X_7_ receptors *in vivo*, due to its low toxicity (Remy et al., 2008), high selectivity (Jiang et al., 2000) and ability to cross the Bbb (Peng et al., 2009). Administration of BBG reduced cord damage and improved motor recovery, associated with reduced inflammatory and glial activation, and infiltration (Peng et al., 2009). The combination of systemically administered Lom, YM872, and BBG have shown good effects in a rodent model of repeated mild traumatic brain injury and comparable to local delivery at improving outcomes following partial optic nerve transection (Mao et al., 2018; Toomey et al., 2019). Therefore, future studies should assess the effects of systemic delivery of blood-brain-barrier permeable Lom and YM872 (Nishiyama et al., 1999) with BBG, following SCI.

## Conclusion

This study provides empirical evidence for the utility of combinatorial ion channel inhibitor treatment regimens to facilitate acute improvements in hindlimb functional recovery, following contusive SCI. We have provided evidence that significant positive changes in early functional recovery and pathophysiology can be achieved acutely with a combinatorial treatment employing Lom, oxATP and YM872 ion channel inhibitors following SCI, including reduced cyst formation and glial reactivity, increased tubulin, reduced demyelination and lipid peroxidation by-products, and changes in mature oligodendrocytes and their progenitors. Very few studies show similar marked improvements in locomotor function at such early time points. Oxidative stress modulation may be beneficial for other acute injury regimes that have shown or show promise, utilizing a combinatorial approach. However, the observed beneficial effects appear to be governed by the method and/or length of delivery of the treatment, highlighting the importance of both an acute therapeutic window and a longitudinal treatment regime. Therefore, future studies should further assess the efficacy of ion channel inhibitor combinations in chronic models of SCI, incorporating therapeutic timing and clinical validity, improved drug delivery mechanisms, bio-activity/availability, and/or blood-brain-barrier permeability properties.

## Data Availability Statement

The datasets generated for this study are available on request to the corresponding author.

## Ethics Statement

The animal study was reviewed and approved by The University of Western Australia Animal Ethics Committee (RA/3/100/1405).

## Author Contributions

RO’H: preparation of the draft manuscript. MF and SH: provided funding and infrastructure support. RO’H, MF and SH: idea conception, experimental design, and data interpretation. RO’H, SS, BF, SR, TS, and CB: data collection and analysis.

## Conflict of Interest

The authors declare that the research was conducted in the absence of any commercial or financial relationships that could be construed as a potential conflict of interest.

## Abbreviations

AMPA: α-amino-3-hydroxy-5-methyl-4-isoxazolepropionic acid
AnkG: ankyrin-G
BBB: Basso, Beattie, and Bresnahan
Ca: calcium
CNS: central nervous system
GAP43: growth-associated protein 43
GFAP: glial fibrillary acidic protein
GluR: glutamate receptor
HNE: 4-hydroxynonenal
K: potassium
MBP: myelin basic protein
Na: sodium
NG2: neural/glial antigen-2
OPC: oligodendrocyte progenitor cell
oxATP: oxidized adenosine triphosphate
P2X: adenosine triphosphate-gated-cation channel family
PDGFα: platelet-derived growth factor subunit alpha
SCI: spinal cord injury
SMI-32: anti-neurofilament H non-phosphorylated
VGCC: voltage-gated calcium channel
YM872: [2,3-dioxo-7-(1*H*-imidazol-1-yl)6-nitro-1,2,3,4-tetrahydro-1-quinoxalinyl] acetic acid monohydrate.
